# Contrast-Enhanced Mammography for Screening Women after Breast Conserving Surgery

**DOI:** 10.3390/cancers12123495

**Published:** 2020-11-24

**Authors:** Jill Gluskin, Carolina Rossi Saccarelli, Daly Avendano, Maria Adele Marino, Almir G. V. Bitencourt, Melissa Pilewskie, Varadan Sevilimedu, Janice S. Sung, Katja Pinker, Maxine S. Jochelson

**Affiliations:** 1 Department of Radiology, Breast Imaging Service, Memorial Sloan Kettering Cancer Center, New York, NY 10065, USA; gluskinj@mskcc.org (J.G.); rossisaccarelli@gmail.com (C.R.S.); davendano@medicos.tecsalud.mx (D.A.); marmarino@unime.it (M.A.M.); almir.bitencourt@accamargo.org.br (A.G.V.B.); sungj@mskcc.org (J.S.S.); pinkerdk@mskcc.org (K.P.); 2 Department of Radiology, Hospital Sírio-Libanês, São Paulo, SP 01308-050, Brazil; 3 Department of Breast Imaging, Breast Cancer Center TecSalud, ITESM Monterrey, 64718 Nuevo Leon, Mexico; 4 Department of Biomedical Sciences and Morphologic and Functional Imaging, University of Messina, 64718 Messina, Italy; 5 Department of Imaging, A.C.Camargo Cancer Center, São Paulo, SP 01509-010, Brazil; 6 Department of Surgery, Breast Service, Memorial Sloan Kettering Cancer Center, New York, NY 10065, USA; pilewskm@mskcc.org; 7 Department of Epidemiology and Biostatistics, Memorial Sloan Kettering Cancer Center, New York, NY 10017, USA; SevilimS@mskcc.org

**Keywords:** breast conserving surgery, breast conservation, contrast enhanced mammography, breast cancer recurrence, breast cancer screening

## Abstract

**Simple Summary:**

Breast cancer survivors are at risk for recurrence, and the early detection of recurrence improves survival. Therefore, imaging surveillance is performed for women who have breast-conserving surgery. The aim of our retrospective study was to compare routine mammography with contrast-enhanced mammography in the screening (asymptomatic) post-treatment setting. We confirmed that when screening women with breast conservation surgery, contrast-enhanced mammography had a higher cancer detection rate (15.4/1000) and positive predictive value of biopsies (42.9%) than full-field digital mammography (6.2/1000 and 37.5%, respectively).

**Abstract:**

To investigate the value of contrast-enhanced mammography (CEM) compared to full-field digital mammography (FFDM) in screening breast cancer patients after breast-conserving surgery (BCS), this Health Insurance Portability and Accountability Act-compliant, institutional review board-approved retrospective, single-institution study included 971 CEM exams in 541 asymptomatic patients treated with BCS who underwent screening CEM between January 2013 and November 2018. Histopathology, or at least a one-year follow-up, was used as the standard of reference. Twenty-one of 541 patients (3.9%) were diagnosed with ipsi- or contralateral breast cancer: six (28.6%) cancers were seen with low-energy images (equivalent to FFDM), an additional nine (42.9%) cancers were detected only on iodine (contrast-enhanced) images, and six interval cancers were identified within 365 days of a negative screening CEM. Of the 10 ipsilateral cancers detected on CEM, four were detected on low-energy images (40%). Of the five contralateral cancers detected on CEM, two were detected on low-energy images (40%). Overall, the cancer detection rate (CDR) for CEM was 15.4/1000 (15/971), and the positive predictive value (PPV3) of the biopsies performed was 42.9% (15/35). For findings seen on low-energy images, with or without contrast, the CDR was 6.2/1000 (6/971), and the PPV3 of the biopsies performed was 37.5% (6/16). In the post-BCS screening setting, CEM has a higher CDR than FFDM.

## 1. Introduction

Survival from breast cancer has improved over the past three decades, with the death rate dropping by 39% from 1989 to 2015, resulting in a growing population of survivors at risk for local recurrence or second primary breast cancer. While rates of local recurrence have also continued to decline secondary to improvements in the locoregional management and effective systemic therapies, women treated with breast-conserving surgery (BCS) still have a risk for in-breast tumor recurrence with rates that vary by tumor subtype and stage [1,2,3,4,5]. Thus, an increasing number of breast cancer survivors, including those who have been treated with BCS, need post-treatment imaging surveillance.

A yearly mammography is recommended by the American Society of Clinical Oncology (ASCO) and American College of Radiology (ACR) guidelines [6,7] for women who have been treated for breast cancer, as it has been demonstrated to improve survival. A meta-analysis of 13 retrospective studies involving 2263 patients demonstrated an improvement in survival when breast cancer recurrence was detected on a mammography when asymptomatic rather than when detected in a physical examination or when there are other symptoms [8]. In another retrospective study of 1044 women, the early detection of asymptomatic breast cancer recurrence improved survival by 27−47% compared with symptomatic recurrence [9]. While early detection is crucial for improved survival, a mammography may be less sensitive in the post-surgical breast compared to the non-treated breast, given that post-treatment changes (including fat necrosis, surgical distortion, metallic clips, and scars) can obscure cancers on screening mammography [10,11,12].

ASCO and ACR guidelines suggest that breast cancer survivors may benefit from additional imaging with magnetic resonance imaging (MRI), particularly survivors with a more than 20% lifetime risk of a second breast cancer or with mammographically dense breast tissue [6,7]. Breast MRI has been shown to increase cancer detection in women who have received BCS [10,13,14]. Nevertheless, it is more expensive and time-consuming than routine mammography and not universally available. Additionally, women who are claustrophobic and those who have pacemakers or other implanted metallic materials cannot undergo MRI [15].

In this setting, contrast-enhanced mammography (CEM) has emerged as a potential alternative method to MRI. CEM is a combined evaluation of anatomy with low-energy (LE) images (equivalent to a routine full-field digital mammogram (FFDM)) and an enhancement of vascularity with recombined images (henceforth referred to as iodine images) [16,17,18]. CEM has been shown to have superior sensitivity to FFDM [17,18,19,20,21,22,23] and can be particularly valuable in women with dense breasts [18,24,25,26,27]. Some studies have shown comparable sensitivity of CEM to MRI [18,28,29].

Despite the promising results of CEM, there are concerns over radiation exposure and the potential for contrast reactions. Studies have shown CEM delivers an average glandular dose that is 20–80% higher than that of FFDM with a per-view average glandular dose range 0.43–2.65 mGy but below the United States Food and Drug Administration limit of 3 mGy specified by the Mammography Quality Standards Act regulations [30,31,32,33]. The adverse reaction rate to intravenous contrast agents is similar to that reported for CT; one meta-analysis found a 0.82% pooled rate of adverse reactions, of which 87% were mild reactions, and only one severe reaction occurred in 14,012 patients [33]. Further studies must be performed to assess the benefits of CEM in different subpopulations. This study was performed to investigate the value of CEM compared with routine FFDM for screening breast cancer patients after BCS.

## 2. Materials and Methods

The study was conducted in accordance with the Declaration of Helsinki, and the protocol was approved by the Institutional Review Board (IRB) of Memorial Sloan Kettering Cancer Center (#16-322) on April 20, 2016. The need for informed consent was waived by the IRB for this single-center retrospective study after determining that the protocol met the requirements as set forth in the regulatory criteria for research approval (45 CFR 46.111 and/or 21 CFR 56.111) and that the investigation was at a low protocol risk level.

### 2.1. Study Cohort

A review of our CEM database and electronic medical records between January 2013 and November 2018 yielded 971 screening CEM examinations performed in 541 consecutive asymptomatic women with a history of breast cancer (invasive carcinoma and/or ductal carcinoma in situ (DCIS)) who had undergone breast-conserving therapy. Radiation therapy was performed in the conserved breast in 503/541 (93%) of the patient cohort.

A portion of this patient cohort has been previously reported in three studies with a different research aim from the present study: 48 women were previously reported in a study comparing background parenchymal enhancement at CEM and breast MRI [34], 61 patients were previously reported in a pilot study comparing CEM and MRI for women at an increased risk for breast cancer [28], and 250 patients were previously reported in a study comparing CEM and low-energy images in screening women at an increased risk of breast cancer [23].

CEM was performed as part of routine clinical care at the request of individual physicians, which was often to screen post-BCS patients with dense breast tissue. For the purpose of this study, patient characteristics that were recorded included age at time of CEM, time from lumpectomy, family history of breast cancer, BReast CAncer (BRCA) gene mutation status, and menopausal status. Patients were categorized as premenopausal, perimenopausal, or postmenopausal (defined as last menstrual period more than 12 months prior to imaging). Mammographic breast density, background parenchymal enhancement, and Breast Imaging Reporting and Data System (BI-RADS) classification were obtained from the first CEM report in the electronic medical record.

### 2.2. Contrast-Enhanced Mammography Technique

CEM was performed using one of the following mammography units: GE Senographe Essential, GE Senographe DS, or GE Senographe Pristina (GE Healthcare, Milwaukee, WI, USA). Patients were given 1.5-mL/kg body weight of Omnipaque 350 (iohexol; GE Healthcare, Shanghai, China), with a maximum injected volume of 150 mL, through a 20-gauge needle using an injector at an injection rate of 3 mL/sec. Once the contrast injection was complete, the patient was positioned for her mammogram. Mammographic imaging was performed with almost simultaneous low (26–30 kVp) and high (45–49 kVp) energy images. Mediolateral oblique and craniocaudal views of each breast were obtained in all patients; those who were within 5 years of their lumpectomy all had at least one additional view of the lumpectomy site as well, per institutional protocol. The low-energy images were interpreted as the FFDM. Postprocessing with a recombination algorithm provided an iodine image that highlighted the areas of contrast enhancement.

### 2.3. Chart Review and Reference Standard

CEM reports, follow-up imaging, biopsy recommendations, subsequent histopathologic results, and at least a one-year mammographic follow-up were reviewed and recorded. Histopathology or at least a one-year follow-up was used as the standard of reference.

BI-RADS 4 or 5 lesions were biopsied with either an ultrasound-guided or stereotactic biopsy when possible. As there was no mechanism for performing a biopsy on findings seen only on iodine images, patients in whom the enhancing abnormality could not be targeted on the ultrasound or stereotactic biopsy were referred for MRI for a potential MRI-guided biopsy. If the lesion was seen on MRI and considered suspicious, an MRI-guided biopsy was performed. However, if the MRI identified a benign etiology, no correlative enhancement or probably benign enhancement either at routine screening or a 6-month follow-up CEM or MRI was performed.

### 2.4. Statistical Analysis

Descriptive statistics were summarized by using frequencies and percentages. As this study only included asymptomatic patients undergoing screening exams, any additional imaging performed with mammography, ultrasonography, and/or MRI was considered as a recall. The recall rate was calculated for CEM low-energy images and iodine images, separately.

To calculate the diagnostic sensitivity and specificity of CEM combining low-energy and iodine images overall, diagnoses were dichotomized by the final BI-RADS as follows: BI-RADS 1, 2, and 3 (no MRI) were classified as a negative result, and BI-RADS 3 (with MRI), 4, and 5 were classified as a positive result. Patients who had a breast MRI prompted by CEM were considered as positive results for the statistical analysis, regardless of MRI findings, because they were referred to MRI for a potential biopsy. Exams were considered as false negatives if the patient was diagnosed with cancer within 365 days following a negative CEM. Screen-detected cancers identified less than 1 year after a negative CEM were considered an interval cancer with respect to CEM.

The sensitivity, specificity, positive predictive value (PPV1), negative predictive value (NPV), and cancer detection rate were calculated on a per-exam basis, along with binomial exact 95% confidence intervals. The positive predictive value of the biopsy performed (PPV3) was calculated per lesion.

Epidemiological parameters such as sensitivity, specificity, negative predictive value (NPV), and positive predictive value (PPV) for CEM were determined using the PROC SURVEYFREQ statement in SAS 9.4 (SAS Institute, Cary, NC, USA). The correlation between successive diagnostic measurements in the same individual was accounted for by using the CLUSTER option available within PROC SURVEYFREQ (SAS Institute, Cary, NC, USA), thereby producing reasonable and robust estimates of the above-mentioned parameters. The cancer detection rate, defined as the number of cancers detected per 1000 screening exams, was also calculated. In order to compare the PPV1 and NPV between CEM (low-energy and iodine images combined) and low-energy images only, a generalized score test statistic proposed by Leisenring [35] was used. In order to compare the sensitivity and specificity of CEM (low-energy and iodine images combined) and low-energy images only, the Exact McNemar [36] test was used. *p*-values < 0.05 were considered as significant. All analyses were performed using SAS software, version 9.4 (SAS Institute, Cary, NC, USA).

## 3. Results

### 3.1. Patient Characteristics

This study included 541 patients with a mean age of 52.5 years at the time of CEM (range, 29−81 years) who underwent a total of 971 exams. The mean time from lumpectomy to the first CEM was 3.3 years (range, 0.2−19.3 years). During the study period, 291 women had one CEM exam, 117 women had two CEM exams, 89 women had three CEM exams, 41 women had four CEM exams, and 3 women had five CEM exams. 

A family history of breast cancer was present in 140/541 (25.9%) women. Six women were BRCA-positive: 1/541 (0.2%) had a BRCA1 and 5/541 (0.9%) a BRCA2 mutation. At the time of breast imaging, 192 women were premenopausal, 220 were perimenopausal, and 129 were postmenopausal, including six women who had oophorectomies.

Breast density was distributed as follows: 51/541 (9.4%) had extremely dense breasts, 420/541 (77.6%) had heterogeneously dense breasts, 68/541 (12.6%) had scattered fibroglandular tissue, and 2/541 (0.4%) had fatty breasts. Background parenchymal enhancement on the first CEM was minimal in 238/541 (44.0%) women, mild in 254/541 (47.0%), moderate in 46/541 (8.5%), and marked in 3/541 (0.5%).

### 3.2. Findings on Low-Energy Images, With or Without Enhancement

**Figure 1:**
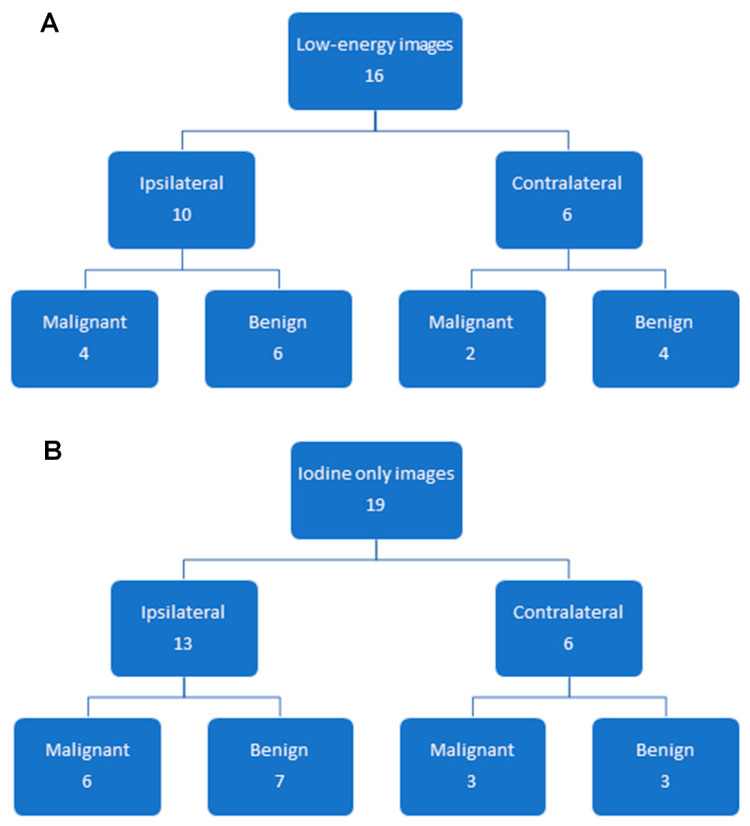
Biopsy of lesions seen on (**A**) low-energy mammograms, with or without enhancement, and (**B**) lesions seen only on iodine images. Interval cancers are not included. Ipsilateral = ipsilateral to the lumpectomy bed and contralateral = contralateral to the lumpectomy bed.

The recall rate on low-energy mammography images, with or without enhancement, was 5.6% (54/971) (Table 1). Of these 54 CEM exams, a breast biopsy was recommended in 16/54 (29.6%) exams, yielding malignancy in 6/16 (37.5%) exams, four in the conserved breast, and two were in the contralateral breast (Table 2 and Figure 1). All six malignant findings were also detected on the iodine images where they were enhanced. The additional imaging work-up was negative in 33/54 (61.1%) exams. Probably benign (BI-RADS 3) low-energy findings were identified in 5/54 (9.3%) exams. Of these five exams, probably benign calcifications were identified in three exams, which were deemed benign on follow-up mammography, and probably benign enhancing asymmetries were identified in two exams, which were deemed benign on follow-up MRI for one exam and follow-up CEM for another exam.

Thus, for the low-energy images, the cancer detection rate was 6.2/1000 (6/971), and the PPV3 of the biopsies performed was 37.5% (6/16).

### 3.3. Additional Findings on Iodine Images

**Figure 2:**
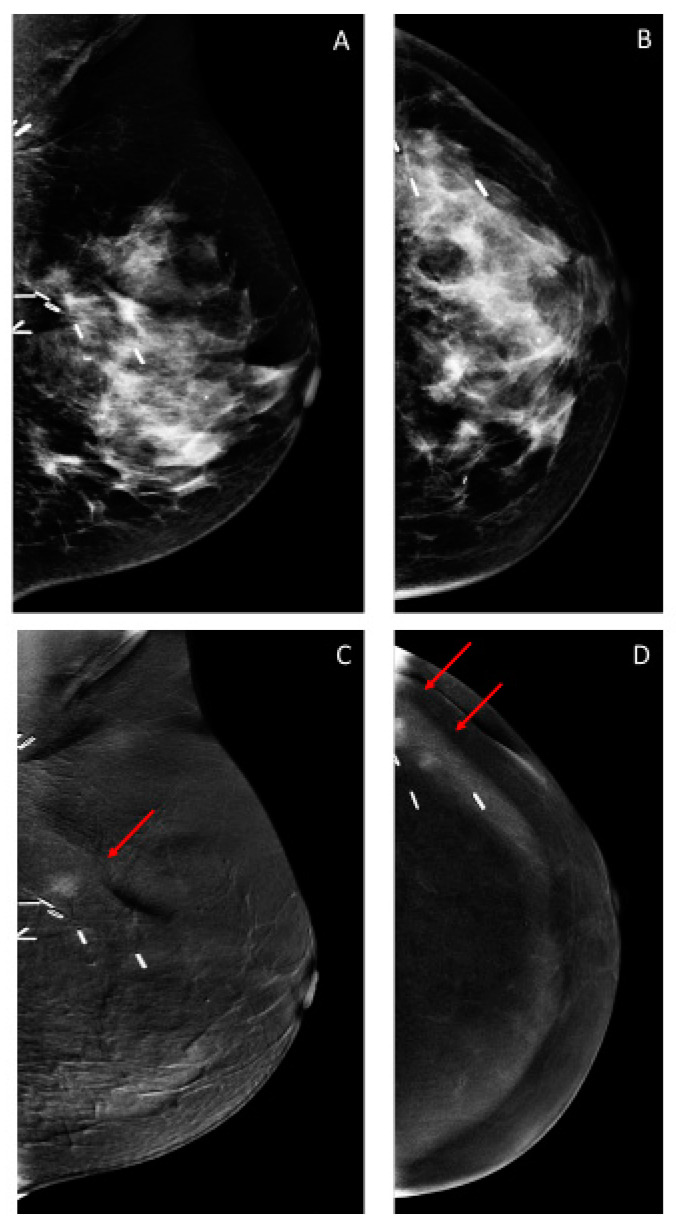
Forty-nine-year-old woman treated with left breast conservation surgery 3 years prior for unifocal invasive ductal carcinoma (IDC) and ductal carcinoma in situ (DCIS). (**A**,**B**) No suspicious findings on low-energy images. (**C**,**D**) Iodine images show two sub-centimeter areas of non-mass enhancement immediately superior and lateral to the surgical clips at the lumpectomy bed (arrows); one lesion is obscured on the mediolateral oblique (MLO) view by an overlying surgical clip. Two sonographic correlates were identified and biopsied, both yielding IDC. The patient was then treated with a mastectomy.

**Figure 3:**
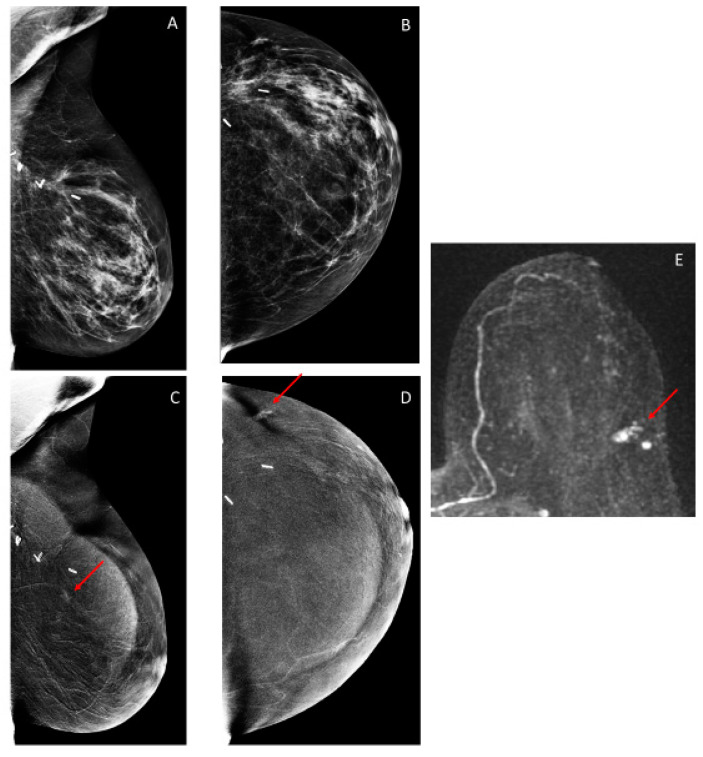
Sixty-six-year-old woman treated with left breast conservation 3 years prior for invasive ductal carcinoma (IDC) and ductal carcinoma in situ (DCIS). (**A**,**B**) Low-energy images show postsurgical distortion but no suspicious findings. (**C**,**D**) Iodine images show clumped non-mass enhancement in the outer central breast (arrows). Targeted ultrasound showed no correlative finding. MRI shows non-mass enhancement spanning 1.3 cm ((**E**) left breast subtraction maximum intensity projection (MIP)); the enhancement was biopsied with MRI guidance, yielding IDC and DCIS. The patient was then treated with a mastectomy.

**Figure 4:**
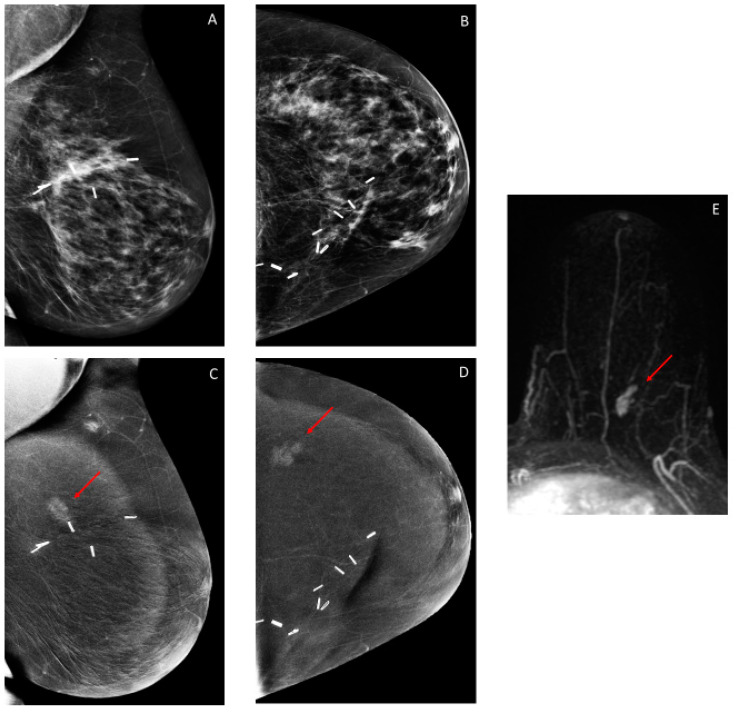
Sixty-five-year-old woman treated with left breast conservation surgery 17 years prior for invasive ductal carcinoma (IDC) and ductal carcinoma in situ (DCIS). Screening CEM shows no suspicious findings on the low-energy images: (**A**) left mediolateral oblique (MLO) and (**B**) left craniocaudal (CC). Iodine images: (**C**) left MLO and (**D**) left CC show focal enhancement in the upper outer quadrant (arrows), new from the prior CEM exam. Targeted ultrasound showed no sonographic correlates. Breast MRI showed correlative enhancement ((**E**) left breast subtraction maximum intensity projection (MIP)); the lesion was biopsied with MRI guidance, yielding DCIS. Left lumpectomy was performed, yielding DCIS. Stable-enhancing skin lesion noted superiorly on MLO images and laterally on subtraction MIP.

The recall rate for additional findings on iodine images (i.e., these findings were not visible on low-energy images) was 4.7% (46/971) (Table 1). Of these 46 CEM exams, a breast biopsy was recommended in 19/46 (43.5%) exams, yielding malignancy in 9/19 (47.4%), six in the conserved breast and three in the contralateral breast (Table 1 and Table 2 and Figure 2, Figure 3 and Figure 4). Additional mammographic and/or ultrasound imaging work-up was negative in 4/46 (8.7%) exams. Probably benign (BI-RADS 3) iodine-only findings were identified on the remaining 23/46 (50%) exams (Table 3). All 23 of these probably benign iodine findings were on the patient’s first CEM. Of these 23 exams, 6 had findings that were deemed probably benign on the CEM, with no MRI follow-up, 11 had MRI findings that were deemed benign on the follow-up MRI, and 6 had findings that were deemed probably benign on the follow-up MRI. In our study population, one BI-RADS 3 exam did not have a follow-up, and the other 22 were found to have no cancer within the following year.

Thus, for iodine images alone, the cancer detection rate was 9.3/1000 (9/971), and the PPV3 of the biopsies performed was 47.4% (9/19).

### 3.4. Overall CEM Results

Additional mammographic views and/or ultrasound were performed following 93/971 (9.6%) CEM exams. A diagnostic breast MRI was prompted by 48/971 (4.9%) exams, which were considered a positive result for the statistical analysis, as MRI was performed for a potential biopsy. In 12/48 (25%) patients, MRI yielded benign findings, in 7/48 (14.6%) patients, MRI yielded probably benign findings, and in the remaining 29/48 (60.4%) patients, an MRI-guided breast biopsy was recommended. No woman had more than one abnormal CEM in this study period.

A breast biopsy was performed in 35 women, yielding 15 cancers (42.9%) (Figure 1 and Table 2). Of the 10 ipsilateral cancers detected on CEM, four were detected on low-energy images (40%). Of the five contralateral cancers detected on CEM, two were detected on low-energy images (40%). Overall, the cancer detection rate for CEM was 15.4/1000 (15/971), and the PPV3 of the biopsies performed was 42.9% (15/35). Thirty-three of the 35 women with biopsies had mammographically dense breast tissue.

### 3.5. Follow-up

A one-year follow-up was available for 917/971 (94.4%) exams. The sensitivity, specificity, PPV1, and NPV were calculated based on these 917 CEM exams and based on the low-energy images alone (Table 4). The CEM exams combining low-energy images and iodine images were much more sensitive and had a slightly higher NPV than the low-energy images alone (sensitivity: 66.7% vs. 27.8%, *p* < 0.01 and NPV: 99.3% vs. 98.5%, *p* = 0.009). The specificity for low-energy images alone was slightly higher than the CEM exams combining the low-energy images and iodine images (specificity: 98.7% vs. 95.8%, *p* < 0.001).

Six patients were diagnosed with an interval cancer within 365 days following a negative CEM. Table 5 shows the features of the interval cancers of these six patients. Three patients had symptomatic cancers that occurred before the next screening interval: two patients presented with palpable abnormalities and one with areolar eczematous changes. The remaining three patients had interval cancers identified in routine screening exams performed within 365 days. Four interval cancers were invasive cancers, and two interval cancers were DCIS (one associated with Paget’s disease). All interval invasive cancers were histologic grade 3, and all interval DCIS were nuclear grade 2.

## 4. Discussion

Following breast cancer treatment, regardless of the surgical approach, women remain at risk for a local recurrence [5]. Hence, in women who have undergone treatment with BCS, ongoing imaging surveillance is necessary. While society guidelines endorse screening with FFDM, its limitations as an anatomic imaging method are well-known [37,38,39,40]. Additionally, BCS creates post-treatment changes that may obscure cancers on a mammography. We therefore evaluated screening CEM compared to FFDM in a population of women who have undergone BCS and found it to be more sensitive at detecting breast cancer.

CEM is the combined interpretation of low-energy images (equivalent to FFDM) and iodine images (analogous to the postcontrast images of breast MRI). CEM has consistently shown improved sensitivity compared with FFDM, particularly in women with dense breasts [25,41,42,43,44,45,46]. A recent publication including 76 women with a history of BCS showed an improved sensitivity of CEM compared with FFDM in the detection of recurrences [47].

We expanded on these prior works by performing a retrospective evaluation of 541 asymptomatic women who had undergone BCS. The addition of intravenous contrast improved the sensitivity from 27.8% to 66.7%. Iodine images identified nine cancers that were not detected on low-energy images, of which eight were invasive. Of the four DCIS lesions identified on CEM, all four were seen on the iodine images (one high nuclear grade, two intermediate grade, and one low grade), whereas three were seen on the low-energy images (the false-negative DCIS on low-energy images was intermediate grade); this suggests that CEM could be useful in the diagnosis of DCIS as well. When comparing the two breasts, 6/10 (40%) ipsilateral cancers were not visualized on low-energy images, and three/five (40%) contralateral cancers were not visualized on low-energy images.

The PPV3 of the biopsies performed was 42.9% in our study. This is comparable to the PPV3 rate of 41% reported in the screening MRI of patients with a personal history of breast cancer [48] and greater than the mean screening mammography PPV3 of 28.6% in the Breast Cancer Surveillance Consortium 2016, which included both average-risk women and those with a personal history of breast cancer [49].

In our study, the abnormal interpretation (recall) rate was 10.3%. This was close to the mean abnormal interpretation rate of 11.6% in the Breast Cancer Surveillance Consortium 2016 screening digital mammography update [45] and within the expected benchmark of 8−25% for screening mammography examinations as suggested by Carney et al. in 2013 [50].

The probably benign (BI-RADS 3) category findings were identified in 2.9% exams, which was below the frequency of reported BI-RADS 3 rates of 6−12% in breast MRI [51,52]. All of the probably benign iodine-only findings were on the patient’s first CEM, suggesting that this rate will decrease once the baseline appearance has been established on CEM. Findings from the iodine images prompted more MRI exams than findings on low-energy CEM images, because there is currently no biopsy mechanism for CEM. A biopsy mechanism for CEM is in the process of being developed, which should reduce the number of MRIs prompted by CEM. We considered all CEMs that prompted a breast MRI as a positive result for the statistical analysis, and therefore, the statistics in Table 4 may change with additional radiologist experience and technologic developments. Benchmark values for CEM are still evolving.

There are several limitations to this study. This was a retrospective, single-institution study where CEM was performed at the request of the ordering physician. There was likely a bias to performing supplemental imaging in women with higher breast density, as most of our patients had mammographically dense breast tissue. Our distinction of the low-energy and iodine image findings was somewhat artificial, as these are two parts of the CEM exam that are always reviewed simultaneously by one radiologist. Additional imaging evaluation of a CEM finding was not standardized amongst radiologists in our group, which led to varied permutations of the imaging work-up, follow-up, and biopsy. We included patients early in our experience with CEM, which may have led to a higher recall rate, more probably benign category findings, and more diagnostic MRI examinations than would happen today after additional experience with CEM.

In women with breast conservation, the post-treatment changes may obscure cancers. Therefore, the sensitivity of low-energy mammography and CEM were evaluated separately in the conserved breast and in the contralateral breast. There was no significant difference in the sensitivities between the two tests comparing the breasts, probably because of our small sample size. This can be a future direction for CEM research.

The findings of our study suggest that CEM in asymptomatic women with a history of breast-conserving surgery improves the cancer detection rate compared to routine mammography alone.

## 5. Conclusions

An annual mammography can decrease mortality in women who have been treated for breast cancer. However, there are limitations of a mammography after breast conservation, as post-treatment changes may obscure cancers. Some have suggested that supplemental imaging, including MRI, might be of value to increase cancer detection. In this study, we demonstrated that contrast mammography, which combines anatomic with vascular imaging, improves the cancer detection rate in patients with breast conservation when compared to mammography, potentially further improving mortality reduction.

## Figures and Tables

**Table 1:** Additional imaging evaluation of findings seen on a low-energy mammography (with or without enhancement) versus on iodine images only.

Imaging Modality	Findings on Low-Energy Images, with or without Enhancement (*n* = 54)	Findings on Iodine Images Only (*n* = 46)
Mammographic views	50	13
Ultrasound	13	42
MRI	12	36

**Table 2:** Histologic characteristics of the biopsied lesions. Interval cancers are not included in this table. DCIS: ductal carcinoma in situ.

Biopsied Lesions	*n*	%
**Malignant**	**15/35**	**42.9**
Invasive ductal carcinoma	8/15	53.3
DCIS	4/15	26.7
Invasive lobular carcinoma	2/15	13.3
Adenosquamous carcinoma	1/15	6.7
**Benign**	**20/35**	**57.1**
Sclerosing adenosis, Usual ductal hyperplasia, Apocrine metaplasia, Periductal inflammation, Dense stromal fibrosis with calcifications in benign epithelium	9/20	45.0
Fibroadenoma/fibroadenomatoid changes	4/20	20.0
Fat necrosis, Scar tissue	5/20	25.0
Atypical lobular hyperplasia	2/20	10.0

**Table 3:** Probably benign imaging findings (Breast Imaging Reporting and Data System or BI-RADS 3) on iodine images only.

Probably Benign Imaging Findings	*n*	%
Focal NME	16/23	69.6
Regional NME	3/23	13.0
Focus	2/23	8.7
Circumscribed enhancing mass Bilateral segmental NME	1/23 1/23	4.3 4.3

NME = non-mass enhancement.

**Table 4:** Comparison of the performance metrics of low-energy images and CEM combining low-energy and iodine images for 917 CEM exams with 1-year follow-up. Interval cancers were included. Recommendations for diagnostic MRI included as a positive result. PPV1: positive predictive value and NPV: negative predictive value.

Performance Metric	Low-Energy Images (95% CI)	CEM (Low-Energy + Iodine Images) (95% CI)	*p*-Value
Sensitivity	27.8% (9.7–53.5)	66.7% (40.9–86.7)	0.01
Specificity	98.7% (97.7–99.2)	95.8% (94.2–97.0)	<0.001
PPV1	29.4% (10.3–55.8)	24.0% (12.9–38.1)	0.53
NPV	98.6% (97.5–99.2)	99.3% (98.6–99.8)	0.009

**Table 5:** Features of interval cancers.

Interval Cancer	Imaging Size, cm	Pathology Size, cm	Pathology Grade	Molecular Subtype of Invasive Cancers	How Interval Cancer was Detected
IDC	1.2	1.3	3	Triple-negative	Palpable
IDC	-	Multiple foci	3	Her2+	Screening ultrasound at 6 months, 29 days
IDC	0.5	0.5	3	Luminal A	Screening MRI at 8 months, 14 days
IMC	1.5	2.0	3	Luminal A	Palpable
DCIS	0.2	1.1	2		Screening mammography at 11 months, 9 days
Paget’s with DCIS	-	1.0	2		Areolar eczematous changes

IDC = invasive ductal carcinoma and IMC = invasive mucinous carcinoma.
